# Draft Genome Sequence of the Streptothricin-Producing Strain *Streptomyces* sp. fd2-tb

**DOI:** 10.1128/genomeA.01277-15

**Published:** 2015-10-29

**Authors:** Biao Tang, Yucong Yu, Xufeng Cen, Yongqiang Zhu, Ruixue Dai, Xianwei Wang, Guoping Zhao, Xiaoming Ding

**Affiliations:** a Collaborative Innovation Center for Genetics and Development, State Key Laboratory of Genetic Engineering, Department of Microbiology, School of Life Sciences, Fudan University, Shanghai, China; b CAS Key Laboratory of Synthetic Biology, Institute of Plant Physiology and Ecology, Shanghai Institutes for Biological Sciences, Chinese Academy of Sciences, Shanghai, China; c Shanghai-MOST Key Laboratory of Disease and Health Genomics, Chinese National Human Genome Center at Shanghai, Shanghai, China

## Abstract

*Streptomyces* sp. fd2-tb can produce streptothricin class antibiotics with broad antimicrobial spectra. To better understand the mechanism of streptothricin biosynthesis and to assess the capacity of this strain in secondary metabolism, we report the draft genome sequence of *Streptomyces* sp. strain fd2-tb.

## GENOME ANNOUNCEMENT

*Streptomyces* sp. strain fd2-tb, which can produce streptothricins (STs), was isolated from a soil sample collected in Shanghai, China. STs can inhibit protein synthesis in prokaryotic cells and show antibacterial, antifungal and antiviral bioactivities (1, 2). STs are used to treat bacterial and fungal diseases in agriculture (3). The 16S rRNA sequence of *Streptomyces* sp. fd2-tb revealed that it belongs to the genus *Streptomyces* using the EzTaxon server (4). The voucher specimen of this actinomycete was deposited at the German Collection of Microorganisms and Cell Cultures (DSMZ) as DSM 100336.

The genome was sequenced using the Illumina HiSeq2500 platform and *de novo* assembly was performed with Velvet version 1.2.03 (5), resulting in 14 scaffolds with 737-fold average coverage. The draft genomic DNA sequences of the paired-end (PE) and mate-pair (MP) DNA libraries were 18,765,858 and 6,698,055 total reads, respectively. The size of the draft genome sequence is 7,646,853 bp, with an average G+C content of 72.03%, and the longest scaffold size assembled is 2,226,054 bp. A total of 7,012 open reading frames (ORFs) were predicted by the RAST server (6) and 76 tRNA genes were predicted by tRNA-Scan program. The coding regions constitute 86.7% of the genome.

Based on the analysis of the antiSMASH tool (7), 28 gene clusters were revealed for secondary metabolites biosynthesis, including 3 siderophores, 6 terpenes, 4 non-ribosomal peptide synthetase (NRPS), 1 mixed terpene/thiopeptide/lantipeptide/NRPS, 1 mixed NRPS/ ladderane, 3 polyketide synthases (PKSs) (1 T1-PKS, 1 T2-PKS, 1 T3-PKS), 2 lantipeptides, 2 bacteriocins, 1 thiopeptide, 2 butyrolactones, 1 melanin, and 2 unspecified clusters. An NRPS gene cluster related to the biosynthesis of streptothricin was revealed with a length of 27,803 bp, including 21 ORFs. Further analysis of the streptothricin is under way.

### Nucleotide sequence accession numbers. 

This whole-genome shotgun project has been deposited in the GenBank/EMBL/DDBJ database under the accession number LBMK00000000. The version described in this paper is the first version, LBMK01000000.
